# Testing the stress-buffering hypothesis of social support in couples coping with early-stage dementia

**DOI:** 10.1371/journal.pone.0189849

**Published:** 2018-01-04

**Authors:** Paul Gellert, Andreas Häusler, Ralf Suhr, Maryam Gholami, Michael Rapp, Adelheid Kuhlmey, Johanna Nordheim

**Affiliations:** 1 Institute of Medical Sociology, Charité – Universitätsmedizin Berlin, Berlin, Germany; 2 Department of Social and Preventive Medicine, University of Potsdam, Potsdam, Germany; 3 Centre for Quality in Care, Berlin, Germany; 4 Department of Family Medicine and Public Health, University of California San Diego, San Diego, United States of America; Coimbra University Hospital Center, PORTUGAL

## Abstract

**Purpose:**

To test whether the negative relationship between perceived stress and quality of life (Hypothesis 1) can be buffered by perceived social support in patients with dementia as well as in caregivers individually (Hypothesis 2: actor effects) and across partners (Hypothesis 3: partner effects and actor-partner effects).

**Method:**

A total of 108 couples (*N* = 216 individuals) comprised of one individual with early-stage dementia and one caregiving partner were assessed at baseline and one month apart. Moderation effects were investigated by applying linear mixed models and actor-partner interdependence models.

**Results:**

Although the stress-quality of life association was more pronounced in caregivers (β = -.63, *p*<.001) compared to patients (β = -.31, *p*<.001), this association was equally moderated by social support in patients (β = .14, *p*<.05) and in the caregivers (β = .13, *p*<.05). From one partner to his or her counterpart, the partner buffering and actor-partner-buffering effect were not present.

**Conclusion:**

The stress-buffering effect has been replicated in individuals with dementia and caregivers but not across partners. Interventions to improve quality of life through perceived social support should not only focus on caregivers, but should incorporate both partners.

## Introduction

There is a high prevalence of family caregiving. In the USA, more than 15 million people provide unpaid care for people with Alzheimer’s disease and other dementias [1]. In Germany, two of three people with dementia are living in the community, many of them receive informal care from their partners [2]. Coping with dementia represents a constant stressor for both the caregiving partner as well as the partner with dementia with negative effect on their quality of life [3]. Perceived social support has shown to buffer the detrimental effects of perceived stress on quality of life [4, 5]. The purpose of this study is to test the stress-buffering hypothesis in couples coping with dementia.

### Stress and quality of life in caregivers and patients

Although caregivers and patients may perceive different levels and sources of distress (e.g., caregivers may struggle with care-related challenges whereas patients perceive stress as a direct result of the disease), in both, findings have been shown negative association with quality of life [6–8]. Despite there are studies that investigate interactions between stress and quality of life across partners [9, 10], the comparison of stress-quality of life relation of caregivers and patients is sparse.

### The stress-buffering role of social support

The stress-buffering hypothesis refers to the perceived availability of social support, which is assumed to eliminate or weaken the negative relationship between perceived stress as a result of a chronic condition on health and quality of life [5, 11]. The stress-buffering hypothesis has been tested in a wide range of studies, although findings remain inconclusive with respect to the specific relational conditions and stressors under which the buffering effect occurs [12–18].

There are hints that the stress-buffering hypothesis might work in people with dementia. Recent meta-analytical results have shown that a lack of social support is associated with incident dementia [19]. In particular, the stress-buffering hypothesis of social support has been discussed as a potential working mechanism, as increased levels of stress are strongly associated with incident dementia [19, 20]. There are hints supporting the stress-buffering hypothesis in individuals with dementia yet it has not been tested explicitly. Bennett et al. [21] found that social support and network characteristics modify the relation of certain measures of Alzheimer’s disease pathology to level of cognitive function.

When looking at family caregivers and spousal caregivers, social support-specific findings indicate that several types of support may relieve physical and mental burden caused by stress for the caregivers [22]. Caregiving itself can be seen as a form of social support provision, potentially functioning as a source of distress, as well as a source of satisfaction [23]. These mechanisms have the potential to affect the strength of stress-buffering in partners with dementia and caregiving partners. In a study with spousal caregivers of dementia patients, social support was moderating the association between caregiver self-efficacy and caregiver subjective burden [24].

### Dyadic approach to the stress-buffering effect

Dyadic approaches to the stress-buffering hypothesis suggest to take the complex interdependence between partners into account (e.g., [25, 26]), especially in the context of caregiving and care-receiving [27]. Perceived social support of the caregiving partner may buffer the negative stress-quality of life relation in patients. One possible explanation of this dyadic stress-buffering effect may be the provision of invisible social support, i.e., the patient is not aware of receiving support [28]. Using data from a daily diary study of support provision and receipt in couples, the authors show that many transactions reported by supporters are not reported by patients [29] underpinning the need for taking a dyadic perspective. Finding by another study have been showing that caregiving partners who are receiving social support by their spouses or by other sources of support are, in turn, more willing to provide adequate social support [26].

Although effects across partners [30] have been studied in dyads of individuals with dementia and their caregiving partners with respect to preventive health behaviors and religious beliefs (e.g., [31, 32]), research testing the stress-buffering hypothesis of social support in couples coping with dementia is warranted.

### Aim of the study

The aim of this study was to test the stress-buffering hypothesis in couples coping with dementia and dementia care.

Hypothesis 1: We hypothesized a negative relationship between perceived distress and quality of life in both partners. Further, it was assumed that the size of this effect is more pronounced in caregivers compared to patients.
Hypothesis 2: The buffering hypothesis was tested in caregiving romantic partners and patients with early-stage dementia within each individual (actor effects) when adjusting for partner effects. The longitudinal association between perceived stress and quality of life was assumed to be moderated by perceived social support; higher values of perceived social support were expected to be related to a less pronounced association of perceived stress and quality of life. This effect was assumed to be stronger in caregivers than in care-receivers.
Hypothesis 3: It was hypothesized that there would be partner buffering effects (partner distress*partner support interaction) or actor-partner buffering effects (actor distress*partner support interaction and partner distress*actor support interaction) although more pronounced in caregivers compared to patients.

## Methods

### Participants and procedure

A total of *N* = 108 patients with dementia and their *N* = 108 caregiving partners (*N* = 216 individuals in 108 couples) from Berlin, Germany (study wave one) and the area surrounding the city (wave two), participated in this prospective study. Measures were taken at baseline and at a one month follow-up (404 valid observations at 2 measurement occasions with 216 at baseline and 188 at follow up). For sample description, see Table 1. The full study design, in which a psychosocial support program has been tested (DYADEM trial, ZQP/ BMBF 01ET1001A), is described in depth elsewhere [33]. Participants were recruited from memory clinics, private practices, nursing services, and other social and medical institutions in and around Berlin. We included couples with one partner diagnosed with dementia as evinced by the National Institute of Aging criteria for all-cause dementia [34], ≥15 in the Mini-Mental Status Examination at baseline (MMSE; [35]), living in the community. Exclusion criteria included severe depression, psychotic disorders, and substance-related and addictive disorders for both partners as well as a dementia diagnosis in the spousal caregiver. Patients and caregivers gave informed consent to participate in this study. In case that the caregiver was the legal guardian of their spouse, they were asked to sign both informed consent letters. The study was approved by the Ethics Committee of the Medical University of Berlin (Charité –Universitätsmedizin Berlin; EA1/215/11).

**Table 1:** Sample Characteristics by Role of Care.

	*Role of Care*	
	*Partner with Dementia* *Mean (SD)*	*Caregiving Partner* *Mean (SD)*
Age in years	74.64 (6.19)	72.04 (6.83)
Gender (*n* women)	42 (38.9%)	66 (61.1%)
Education in years	15.06 (14.82)	14.06 (12.19)
Quality of life (WHO-QoL-BREF/ QOL-AD)	36.42 (5.68)	96.72 (12.14)
Perceived social support (F-SozU-14)	54.35 (7.76)	53.48 (11.28)
Perceived distress (PSS)	9.81 (5.26)	12.77 (4.35)
Depressive symptoms (GDS)	5.51 (2.36)	4.74 (2.07)
Functional disability (ADL)	88.80 (15.21)	-
Cognitive functioning (MMSE)	22.81 (4.33)	-

*Note*. *N* = 216 participants. Baseline values were reported. Activities of Daily Living (ADL), Mini-Mental Status Examination (MMSE), and Brief Quality of Life Instrument of the World Health Organization (WHO-QoL-BREF) used in caregivers only. Quality of Life – Alzheimer’s Disease (QOL-AD) used in partners with dementia only. Perceived Social Support Questionnaire (F-SozU-14). Perceived Stress Scale (distress subscale) (PSS). Geriatric Depression Scale (GDS).

We initially contacted *N*_*D*_ = 212 dyads, of whom 68 declined to participate or did not respond. Reasons indicated by those who declined to participate include: lack of acceptance of the disease (*n*_*D*_ = 7), nursing home transmission or death (*n*_*D*_ = 8), being overburdened (*n*_*D*_ = 13), severe somatic diseases in one or both partners (*n*_*D*_ = 11), and other, unstated reasons (*n*_*D*_ = 29). 26 dyads were excluded because of dementia severity (MMSE < 15), 6 dyads because the assumed dementia could not be diagnostically confirmed, 3 dyads because of alcohol abuse in one of the spouses and 1 dyad because of nursing home transmission. We thus enrolled 108 dyads in our study. A total of 76 of the included individuals were suffering from Alzheimer’s disease, 13 from a vascular dementia, and three had dementia in Parkinson’s disease. Four individuals had a diagnosis of a dementia in other specified diseases classified elsewhere and in 12 individuals the dementia diagnosis was not otherwise specified. Data were collected from November 2011 to April 2013 (wave one) and March 2014 to April 2015 (wave two) by specially trained research assistants at participants’ homes.

### Measures

For means (*M*) and standard deviations (*SD*s) of all variables under study broken down by partner with dementia versus caregiving partner, see Table 1.

*Perceived stress* was used as a time-varying level-1 variable measured with the stress subscale of the Perceived Stress Scale (PSS) and served as a predictor of quality of life in all models [36]. PSS has previously been used with dementia patient caregivers [37, 38].

*Perceived social support* was used as a time-varying level-1 variable was measured with the 14 item questionnaire F-SozU-14 [39]. F-SozU is a unidimensional measure not specific to a certain source of support (partner, friends, or family). In all of our models, F-SozU served as a buffering variable when used as an interaction term (stress*social support).

*Quality of life* was used as a time-varying level-1 variable is an outcome in this study. The caregiving partner’s quality of life was measured by the WHO-QoL BREF of the World Health Organization [40]. In partners suffering from dementia, the QOL-AD was applied [41]. In a first step, WHO-QoL BREF and QOL-AD were transferred to the same 0–100 scaling ((raw score—lowest possible raw score/ possible raw score range)* 100). Then, in order to allow comparison across partner roles, values have been z-standardized across all individuals [42].

*Depressive symptoms* was used as a time-varying level-1 variable and served as a covariate, measured by the GDS-SF-15 (Geriatric Depression Scale– 15 items-Short Form; [43]). The GDS-SF-15 uses a dichotomous yes/no response format. Total score ranges from 0–15, with a score of 5 or more indicating probable depression. It has been shown [44] that the GDS-SF-15 can be reliably used in individuals with early-stage dementia.

*Age* and *gender* were used as an individual-level-2 variable and served as covariates in the models. Age ranged from 55 to 89 years of age with a mean age of 74.64 (*SD* = 6.19) years in partners with dementia and 72.04 (*SD* = 6.83) years in caregivers. Due to the couple-centered study design, there where exactly 108 men and 108 women included into the study.

*Role of care* was introduced as a level-2 variable on an individual level and served as grouping variable in the analysis of hypothesis 2 and 3. A total of 44 individuals with dementia and 66 of the caregivers were women (66 individuals with dementia and 44 caregiving partners were men).

*Functional disability* (Barthel index) served as a covariate and was used as a level-3 variable on the couple level and is related to the ability of daily self-care activities such as bathing, dressing, grooming, or work [45]. Both partners’ quality of life, the patient as well as the caregiver was regressed on the activities of daily living (ADL) score of the patient, indicating the dementia-related burden for the whole couple. Caregivers were indicating the functional disability status of their partners.

*Cognitive functioning*, such as memory, attention, and language, was assessed with the Mini-Mental State Examination (MMSE; [35]) and served as a covariate defined as a couple-level variable similarly to ADL (again, level-3 or couple level). Higher values of MMSE represent better cognitive functioning.

### Statistical analysis

Before going into statistical details, we will explain the main structure in a straightforward manner. In ordinary statistical models, observations are assumed to be independent from each other. Since this assumption of independence of observations is clearly violated in research that investigates individuals in interdependent relationships, the models should take the non-independence of this data into account. The Actor – Partner Interdependence Model (APIM) integrates conceptual and statistical considerations of interdependence in two person relationships [30]. Moreover, this model was developed not only with non-independence in mind, but also to allow for testing couple-related hypotheses based on the interdependences across partners. The two-intercept model gives distinct coefficient estimates for the two partners in a single model, allowing for the collection of the actor-partner dependencies for each partner role separately – for partner with dementia separately from the caregiving partner. To add another level of complexity, non-independence also applies for repeated measurements within an individual, which we have accounted for by using linear mixed modelling [46].

All data analyses were conducted using MIXED procedure in SPSS v22 (IBM, 2013). In order to take the dependency within the data structure into account linear mixed models with random intercepts were used. Measurement occasions (time 1, time 2) were nested in individuals. The dependencies across partners within the same couple were modelled applying two-intercept models. The two-intercept linear mixed models with random intercept were established by creating an effect coded variable with partner with dementia = -1 and caregiving partner = 1 and by multiplying each predictor variable with the effect coded partner variable. Thereby, patients and caregiving partners got separate intercept and coefficient estimates within the same model. More details can be found in Kenny, Kashy, & Cook, Chapter 7 [42]. Linear mixed models with Restricted Maximum Likelihood estimator (REML) accommodate all available data (*N* = 216 individuals; 108 couples), retaining cases for which missing data are present, and will typically yield unbiased estimates if data are missing at random. A time variable was introduced into the models, centered at the initial status; therefore, the intercept of the model was interpreted as participant’s evaluation of the quality of life at baseline.

Within the linear mixed models framework, actor-partner interdependence models have been specified for hypotheses testing to take the interdependence between partners into account when analyzing this data [30]. Research that applies a dyadic approach on couple data distinguishes between actor and partner effects. Actor effects refer to associations of predictors and outcome variables within each partner or individual. In contrast, partner effects refer to when the predictor variable is measured in one partner and the outcome is measured in the other.

In order to test the Hypothesis 2, an actor distress*actor support interaction term was established and would indicate that the moderation of the stress-quality of life association by perceived social support would be present in the respective group (actor-buffering effect in patients as well as in caregivers).

For Hypothesis 3 (partner buffering effect and actor-partner stress-buffering effect), each individual’s quality of life was regressed on his or her own predictor values (actor effect; see Hypothesis 2), on his or her partner’s predictor values (partner effect), and on the interaction terms partner distress*partner support, actor distress*partner support, and partner distress*actor support simultaneously. These actor and partner effects moderated by the partner’s moderator variable were also called “mixed moderator effects” and have been described in Garcia, Kenny, and Ledermann [47] in more detail. Post hoc contrast tests for differences between patients and caregivers were performed testing whether the coefficient of the patient minus the coefficient of the caregiver is statistically different from zero.

All continuous predictor variables were defined as time-varying variables and grand-mean centered, whereas gender (men = 0, women = 1) and role of care (partner with dementia = 0, caregiving partner = 1) were dummy coded prior to analysis. Age, gender, depressive symptoms, functional disability, and cognitive functioning served as covariates in the final model.

## Results

In a null model without predictors (not displayed), the proportion of couple variance of the total variation was 3.4% (intra class correlation coefficient [ICC]). Bivariate associations were in the expected direction (see Table 2). Quality of life was positively related to perceived social support (β = .28, *p*<.001) and negatively related to perceived distress (β = -.38, *p*<.01).

**Table 2:** Standardized bivariate associations among study variables.

	*Variables*	*1*	*2*	*3*	*4*	*5*	*6*	*7*	*8*	*9*	*10*
1	Time	-									
2	Quality of life	-.19***	-								
3	Perceived social support (actor)	-.07	.28***	-							
4	Perceived stress (actor)	-.02	-.38***	-.13**	-						
5	Partner social support	-.08	-.01	-1.00	.02	-					
6	Partner stress	-.02	-.05	.04	-1.00	-.13*	-				
7	Age	-	.01	.01	.13*	-.04	-.07	-			
8	Gender ^a^	-	.05	-.02	-.15**	.05	.15*	-.26**	-		
9	Depressive symptoms	.05	-.17**	.01	.18***	.03	.10	.01	.04	-	
10	Functional disability	-.16***	.32**	.06	-.13*	.06	-.13*	.15**	-.002	-.21**	-
11	Cognitive functioning	-.24***	.08	.04	.03	.06	.03	-.10*	-.003	.05	.33**
12	Condition	-	.04	.04	-.02	.03	-.05	.08	-	-.03	-.09
13	Region	-	-.01	-.01	.06	.00	.06	-.04	-	.13*	-.03

*Note*. Standardized bivariate associations were accounted for the correlated data structure using linear mixed models. 404 observations, *N* = 216 participants, 108 couples;

^a^ Gender is coded 0 = men 1 = women; Region is coded 0 = suburban or rural 1 = urban. Condition is coded 0 = control 1 = psychosocial group. Functional disability was assessed in partners with dementia via activities of daily living (ADL) scale. Cognitive functioning (memory, attention, and language) was assessed in partners with dementia via Mini-Mental Status Examination (MMSE). Association between two dichotomous variables were not reported.

*p < .05;

** *p* < .01;

***p < .001.

The first hypothesis was confirmed (see Table 3). The stress-quality of life association was significant in patients (β = -.31, *p*<.001) as well as in caregivers (β = -.63, *p*<.001) and more pronounced in caregivers compared to patients (difference test: β = -.32, *p*<.001).

**Table 3:** Actor-partner interdependence model (APIM) of perceived distress-social support interaction (buffering effect) regressed on health-related quality of life.

Variable	*Two-Intercept Model without covariates*			*Two-Intercept Model with covariates*		
	*Partner With Dementia* ^a^	*Caregiving Partner* ^a^	Contrast test for differences of coefficients across care roles	*Partner With Dementia* ^a^	*Caregiving Partner* ^a^	Contrast test for differences of coefficients across care roles
	β *(SE)*	β *(SE)*	β *(SE)*	β *(SE)*	β *(SE)*	β *(SE)*
Intercept	-.04 (.08)	.39 (.08)***	.43 (.11)***	-.40 (.17)*	.28 (.17)**	.67 (.23)**
Time	-.21 (.08)*	-.29 (0.8)***	-.09 (.11)	-.13 (.08)	-.27 (.08)**	-.14 (.11)
*Actor-Effects* (time varying)						
Actor perceived distress	-.37 (.06)***	-.64 (.07)***	-.26 (.09)**	-.31 (.06)***	-.63 (.06)***	-.32 (.09)***
Actor perceived social support	.15 (.09)*	.17 (.06)*	.02 (.11)	.21 (.08)**	.17 (.05)**	-.04 (.10)
Actor distress*actor support interaction	.15 (.07)*	.13 (.06)*	-.02 (.09)	.14 (.06)*	.13 (.06)*	-.02 (.09)
*Partner-Effects* (time varying)						
Partner perceived distress	.04 (.07)	.04 (.06)	.01 (.10)	.04 (.07)	.02 (.06)	-.01 (.09)
Partner perceived social support	.03 (.06)	-.03 (.07)	-.02 (.11)	.01 (.06)	-.05 (.08)	-.06 (.11)
Partner distress*partner support interaction	.03 (.06)	.11 (.07)		.00 (.06)	.12 (.07)	.11 (.09)
*Actor-Partner-Interaction Effect* (time varying)						
Actor distress*partner support interaction	-.06 (.05)	.09 (.08)	.15 (.09)	-.04 (.05)	.08 (.08)	.12 (.09)
Partner distress*actor support interaction	.09 (.08)	.06 (.05)	-.03 (.10)	.01 (.08)	.05 (.05)	.04 (.09)
*Covariates*						
Depressive symptoms (time varying)				-.26 (.06)***	.04 (.07)	.29 (.09)***
Age				.05 (.07)	.02 (.07)	-.03 (.10)
Gender ^b^				.07 (.13)	.18 (.15)	.11 (.20)
Functional disability of the patient regressed on both partners quality of life				.20 (.07)**	.08 (.07)	-.12 (.09)
Cognitive functioning of the patient regressed on both partners quality of life				-.02 (.07)	.01 (.07)	.03 (.10)
Condition				.22 (.13)	.00 (.13)	-.22 (.18)
Region				.36 (.15)*	.06 (.15)	-.30 (.21)
Estimate of variance by care role	.42 (.09)***	.33 (.07)***		.25 (.06)***	.32 (.07)***	
Correlation between variances of care roles (CHS rho)	.25 (.14)			.14 (.16)		
Time variance	.04 (.08)			.02 (.07)		
Residual variance	.23 (.04)***			.24 (.05)***		
-2 Restricted Log Likelihood	863.68			847.91		
*AIC* ^c^	873.68			857.91		

*Note*. All continuous variables were grand-mean centered prior to analyses (z-standardization with mean = 0 SD = 1) and can be interpreted like standardized β coefficients.

^a^ Role of care was effect coded -1 = Partner with dementia 1 = Caregiving partner. Functional disability was assessed in partners with dementia via activities of daily living (ADL) scale. Cognitive functioning (memory, attention, and language) was assessed in partners with dementia via Mini-Mental Status Examination (MMSE).

^b^ Gender is coded 0 = Men 1 = Women. Region is coded 0 = suburban or rural 1 = urban. Condition is coded 0 = control 1 = psychosocial group.

^c^ *AIC* = Akaike’s Information Criterion; in nested models lower AIC values indicate better fit. CHS rho = test of non-independence across partners.

*p < .05;

** *p* < .01;

***p < .001

**Fig 1:**
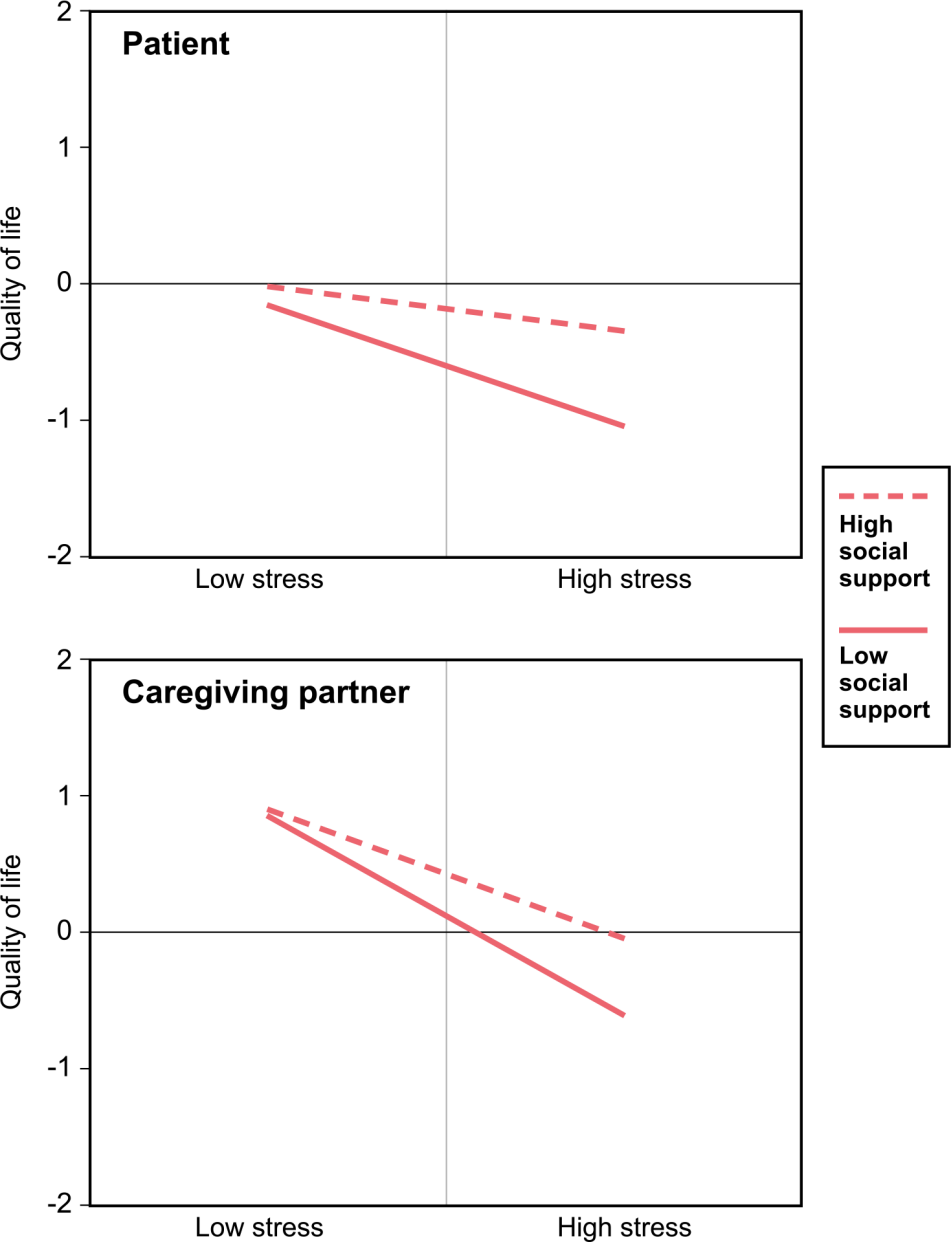
The stress-buffering effect of perceived social support on health-related quality of life separately for patients and their caregiving partners (Hypothesis 2). The solid line with diamond symbols represents predicted values from the adjusted mixed model of low perceived social support (one standard deviation below the mean); the dashed line with square symbols represents predicted values of high perceived social support (one standard deviation above the mean). Low perceived distress refers to values of one standard deviation below the mean; high perceived distress refers to values one standard deviation above the mean. All variables were centered with a mean value of zero (z-standardization).

Confirming the second hypothesis (actor effects), the buffering effect was present (Table 3; Two-intercept model). This was indicated by the significant stress*support interaction coefficient of β = .14 (*p*<.05) and of β = .13 (*p*<.05), for the patient and the caregiving partner, respectively. The negative association of perceived stress and quality of life became weaker the more social support an individual perceived (Fig 1 and Table 2). Yet, the stress*support interaction was equally strong in both partners (difference test: β = -.02, *p*>.05). The magnitude of the coefficient of depressive symptoms has shown to vary across partner groups (difference test: β = .29, *p*<.001) with a strong negative association in patients (β = -.26, *p*<.001) and no association in caregiving partners (β = .04, *p*>.05). Moreover, patients of couples living in urban regions showed higher levels of quality of life (β = .34, *p*<.05) compared with couples in suburban or rural areas.

Regarding the third hypothesis (actor-partner and partner-partner interaction; Table 3), within APIM, the buffering effect did not occur as an actor distress-partner social support interaction effect in patients (β = -.04, *p*>.05) that is caregiver’s perceived social support did not buffer the patient’s negative distress-quality of life association. The same non-significant result was found for actor-partner interaction in caregivers (β = .08, *p*>.05). All other possible actor-partner and partner-partner interaction turned out to be non-significant (*p*>.05).

## Discussion

The aim of this study was to test the stress-buffering hypothesis in couples coping with dementia and dementia care. We investigated the function of the buffering effect in patients with dementia and their caregiving romantic partners and whether this buffering effect occurs in dyads on an individual level and across partners.

Confirming the first hypothesis within the actor-partner interdependence framework, the perceived distress was negatively associated with quality of life in patients as well as in caregivers. Further, the magnitude of the relation was stronger degree in caregivers compared to patients. This finding adds to the literature that mostly focuses on either the patients or the caregiver [27]. Different levels and sources of distress in patients and caregivers might explain the differences in the magnitude of the stress-quality of life relation between caregivers and patients. Caregivers may struggle with care-related demands [6, 48] whereas patients perceive stress as a direct result of the disease [8] although both partners also share sources of distress such as daily hassles related to care or relationship problems.

Regarding the second hypothesis, the stress-buffering effect was confirmed in both partners. The negative association of perceived distress and health-related quality of life was less pronounced in individuals with high levels of perceived social support compared with individuals with low social support. The buffering effect was equally present in partners with dementia and in caregiving partners. As studies of individuals with dementia have shown, increased levels of perceived social support might not only result in better quality of health – which has been shown by the present findings, but increased social support might also be a source of regular brain stimulation [21], relaxation and enjoyment, and contribute to healthy behavior [19]. In caregiving partners, the buffering effect might result in improved quality of life through less behavioral disturbances in the care-receiving partner, lower caregiver burden [22, 49], and improved couple communication [50, 51].

The third hypothesis could not be confirmed for any of the partner and actor-partner effects. In the present study, the buffering effect occurred as an actor effect within individuals only (Hypothesis 1) rather than from one partner to his or her counterpart. The expected actor-partner moderation effect that is one partner who perceive high levels of social support would buffer the stress-quality of life association in their counterpart could not be supported. Our results are in line with literature that found stronger actor effects than effects between partners (e.g., [52])–though other studies found stronger partner effects (e.g., [31]). Psychologically, the strong actor-buffering effects (and the missing partner effects and actor-partner moderation effects) might refer to the phenomenon that one’s own perception of social support is likely to be a more visible and important factor of one’s own quality of life compared with partners perceptions, which might be channeled through their own perceptions [53, 54].

### Implications of the present findings

The present findings have implications for clinical interventions. Since we found the stress-buffering effect to be present in patients as well as in spousal caregivers, support groups, family therapy, and counselling should focus on both partners [50]. Meta-analytical results confirm that addressing both partners, the caregiver as well as the care-receiver leads to more effective interventions compared with interventions that target the patient only [55, 56]. Berg and Upchurch [26] emphasized that interventions may provide not only greater facilitation for the patient but also much needed intervention for the distressed spousal caregiver. Our findings extend the current literature on dementia caregiving by explicitly testing effects across partners. As partner effects and actor-partner moderation effects could not be detected in the present data, more research is needed to specify whether and under which conditions possible effects occur across partners.

### Limitations

We assessed perceived social support in a univariate manner. However, our method has some limitations. Distinguishing between different categories of social support might reveal differential patterns. For instance, Wolff et al. (2014) found differential buffering effects for emotional and instrumental social support on health complaints in older adults [13]. Moreover, we focused on individuals within relationships; thus, a constant potential source of social support was available. Future studies should replicate the stress-buffering effect in single or widowed individuals with dementia and their professional caregivers. We included patients with mild-to-moderate dementia across different types of dementia, which might have increased heterogeneity of our sample. However, we did not find significant differences in all variables under study between those with Alzheimer’s disease compared with those diagnosed with other types of dementia, with exception of functional disability, where those with Alzheimer’s showed better functioning than those with other types of dementia (t[3.0], p = .004; Alzheimer’s Mean = 92.0; Other’s Mean = 82.7). In this instance, heterogeneity still may be present to a degree. In our final model, we adjusted for differences in functional disability; future studies may analyze certain types of dementia separately. Nevertheless, there are many high-quality studies analyzing data of different types of dementia, such as Alzheimer’s disease and vascular dementia (for a review of literature, see [57]). In a comparative study, Perri et al. (2014) found that behavioral symptoms in dementia were mostly unrelated to the specific type of dementia or specific cognitive deficit. These symptoms constitute an important strain, especially on the caregiving partners of all dementia patients, which we addressed with our study [58]. These symptoms constitute an important strain especially for the caregiving partners of all dementia patients, which we addressed with our study. Finally, our sample was limited in dementia severity. Stressors and coping mechanisms are likely to be different in more severe and later stages of dementia.

Nevertheless, we found a stress-buffering effect present in both partners as well as actor effects revealed to be strong whereas partner effects turned out to be not present. This study adds to the literature of buffering effects in different populations and suggests that interventions intended to foster social support should not only target caregivers, but ultimately target both partners – the partner with dementia and their caregiving partner.
